# Acute care research competencies for clinical research professionals

**DOI:** 10.1017/cts.2020.38

**Published:** 2020-04-13

**Authors:** Stephanie Schuckman, Lynn Babcock, Cristina Spinner, Opeolu Adeoye, Dina Gomaa, Timothy Pritts, Brett M. Kissela, Christopher J. Lindsell, Jacqueline M. Knapke

**Affiliations:** 1 Center for Clinical and Translational Science and Training, University of Cincinnati, Cincinnati, Ohio, USA; 2 Division of Emergency Medicine, Cincinnati Children’s Hospital Medical Center, Cincinnati, Ohio, USA; 3 Department of Regulatory Submissions, Medpace, Cincinnati, Ohio, USA; 4 Division of Neurology and Rehabilitation, University of Cincinnati, Cincinnati, Ohio, USA; 5 Division of Emergency Medicine and Stroke Team, University of Cincinnati, Cincinnati, Ohio, USA; 6 Division of Trauma Surgery and Critical Care, University of Cincinnati, Cincinnati, Ohio, USA; 7 Department of Biostatistics, Vanderbilt University Medical Center, Nashville, Tennessee, USA

**Keywords:** Acute care research (ACR), special interest competencies, competency-based education, clinical research professionals (CRPs), Clinical Translational Science Award (CTSA)

## Abstract

**Introduction:**

Acute care research (ACR) is uniquely challenged by the constraints of recruiting participants and conducting research procedures within minutes to hours of an unscheduled critical illness or injury. Existing competencies for clinical research professionals (CRPs) are gaining traction but may have gaps for the acute environment. We sought to expand existing CRP competencies to include the specialized skills needed for ACR settings.

**Methods:**

Qualitative data collected from job shadowing, clinical observations, and interviews were analyzed to assess the educational needs of the acute care clinical research workforce. We identified competencies necessary to succeed as an ACR-CRP, and then applied Bloom’s Taxonomy to develop characteristics into learning outcomes that frame both knowledge to be acquired and job performance metrics.

**Results:**

There were 28 special interest competencies for ACR-CRPs identified within the eight domains set by the Joint Task Force (JTF) of Clinical Trial Competency. While the eight domains were not prioritized by the JTF, in ACR an emphasis on Communication and Teamwork, Clinical Trials Operations, and Data Management and Informatics was observed. Within each domain, distinct proficiencies and unique personal characteristics essential for success were identified. The competencies suggest that a combination of competency-based training, behavioral-based hiring practices, and continuing professional development will be essential to ACR success.

**Conclusion:**

The competencies developed for ACR can serve as a training guide for CRPs to be prepared for the challenges of conducting research within this vulnerable population. Hiring, training, and supporting the development of this workforce are foundational to clinical research in this challenging setting.

## Introduction

Acute care research (ACR) is characterized by the need for rapid participant identification and conduct of study procedures, often in emergent and sometimes life-threatening situations. As in other settings, clinical research professionals (CRPs) perform many pivotal duties in ACR, including study coordination and site support, project management, and regulatory compliance [1]. However, unlike traditional CRPs, ACR-CRPs must perform these duties in stressful environments with time constraints, such as in the pre-hospital setting (e.g., accident scenes and ambulances), emergency department (ED), or intensive care units (ICUs), as opposed to scheduled visits either in person or over the phone. ACR-CRPs are tasked with managing the challenges of recruitment of a vulnerable population presenting with a new or worsening condition at unscheduled times. Although general competencies for research coordinators have been described in the literature [1,2-4], the competencies that are unique to ACR have not been detailed in the literature nor outlined by governing bodies, leaving a major gap for supporting the development of this essential research workforce. This gap is being addressed by the Cincinnati Acute Care Research Council (ACRC).

The ACRC was formed in 2015 by the Center for Clinical and Translational Science and Training (CCTST), a Clinical Translational Science Award hub at the University of Cincinnati (UC). A complete list of ACRC leadership and partners is provided in the appendix. The purpose was to create an inclusive community of researchers engaged in pre-hospital, emergency, and critical care settings with a common focus on accelerating ACR by reducing systems barriers and generating economies of scale through process improvement, resource sharing, and development of best practices. Our Academic Health Center is comprised of three large freestanding hospitals: the Cincinnati Veterans Administration Medical Center (VAMC), the University of Cincinnati Medical Center (UCMC), and Cincinnati Children’s Hospital Medical Center (CCHMC), such that the ACRC spans all ages and communities. Both UCMC and CCHMC operate Level 1 Trauma Centers. The ACRC has partners from 14 diverse adult and pediatric disciplines, local institutional review boards, and investigational pharmacies.

From the onset, the ACRC recognized the importance of the highly trained CRPs. They are the “boots on the ground” that ensure rapid and proper research subject identification, consent, enrollment, and conduct of study procedures. Because of the constraints of ACR, the ACRC determined that ACR-CRPs required additional competencies beyond those of CRPs engaged in other types of clinical research. Moreover, developing such competencies is timely given the current focus on CRP workforce development [5]. In 2014, the Joint Task Force (JTF) for Clinical Trial Competency produced a single set of CRP competencies called the “Harmonized Core Competency Framework for the Clinical Research Professional.” They defined 51 competency statements addressing CRP knowledge, skills, and attitudes under 8 scientific domains: 1) Scientific Concepts and Research Design, 2) Ethical and Participant Safety Considerations, 3) Medicines Development and Regulation, 4) Clinical Trial Operations, 5) Study and Site Management, 6) Data Management and Informatics, 7) Leadership and Professionalism, and 8) Communication and Teamwork [1]. In 2017 and 2018, the JTF framework was refined into 48 competency statements [4] and “leveled,” adding competency levels for each statement [2,3]. These frameworks provide an excellent foundation on which to develop additional competencies to meet the unique needs of CRPs involved in ACR. The ACRC therefore sought to develop an ACR-CRP competency framework to guide the creation of a multipronged translational ACR workforce development program that could include standardization of competency-based curricula and career performance expectations.

## Study Design and Methodology

The ACR-CRP competencies were developed using a dynamic approach taken from instructional systems design, involving iterative phases called ADDIE – *Analyze, Design, Develop, Implement, and Evaluate* [6]. Continual feedback is gathered at each phase to produce a work product, which is then used to guide the next stage of the development of educational materials. To avoid confusion in nomenclature when communicating within our transdisciplinary research team, we chose to use “Assess” rather than “Analyze,” and for the purposes of this study, we focused on the first three ADDIE elements only (*Assess-Design-Develop*), with *Implementation* and *Evaluation* to come in future work. To bound the construct of ACR, the ACRC has defined it based on its unique challenges of occurring within 24 h of an unscheduled health event, specifically [7]:

1. *Need to interface with patients 24/7, within minutes to hours of their illness or injury*,
2. *Inability to schedule patients for recruitment*,
3. *Frequent inability to directly consent patients due to patient care and family considerations that surround a catastrophic health event*,
4. *Movement of patients throughout the health care system (Emergency Medical Services/Prehospital, EDs, Surgery, ICUs, Regular Patient Care Units)*,
5. *Population at higher risk of health disparities and health failure due to using EDs as a primary resource of care.*

**Fig. 1.**
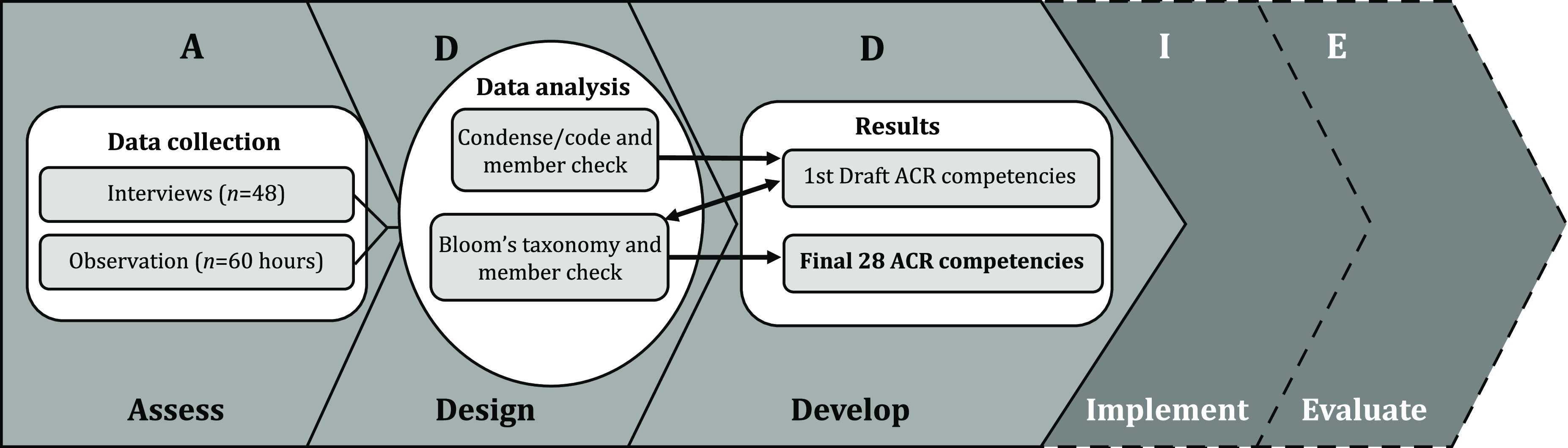
Assess, Design, Develop, Implement, and Evaluate framework and study methods.

Fig. 1 provides an overview of the methodology; details of the methods are described below. Briefly, in the data collection phase, data were gathered using multiple strategies to *Assess* the problem and determine unique ACR-CRP characteristics that would be used to inform the design of the competencies. In the data analysis phase of the project, repeated *Assess, Design*, and *Develop* processes were conducted to produce the final product: ACR-CRP competencies. Throughout data analysis, the research team employed member checking [8], a qualitative technique that allows participants to comment on initial findings, ensuring credibility. Subsequent amendments were made to the work product based upon the feedback gathered.

### Recruitment and Sampling

Subjects were recruited, interviewed, and observed to generate data to assess the ACR landscape and determine the types of behaviors, knowledge, and skills that are unique to ACR-CRPs. Two stages of recruitment were used to gather a stratified sample that achieved representative proportions of the stakeholder population by institutional affiliation, professional position, and range of training and career experience. Participants included members from across the Cincinnati Academic Health Center involved in the ACRC as well as affiliated Advisory Councils – ACR-CRPs, ACR-Regulatory Professionals (ACR-RPs), and CCHMC Research Participant Advisory Council (RPAC). In the first stage of recruitment, a convenience sample of ACR stakeholders was identified by the ACRC for approach. They were typically colleagues or staff selected to provide a broad representation of ACRC member constituencies. In the second stage of recruitment, snowball sampling connected additional subject matter experts as identified by participants in the first stage. All participants were invited to participate in all aspects of the study; whether a participant contributed via interview or observation or both was up to the individual. Participants received no financial incentive for their participation. Informed consent was not required; in consultation with the Institutional Review Board it was determined that this study satisfied Category 2 (Anonymous Educational Tests, Surveys, Interviews, or Observations) as defined by 45 Code of Federal Regulations 46.101(b).

### Data Collection Methods

**Fig. 2.**
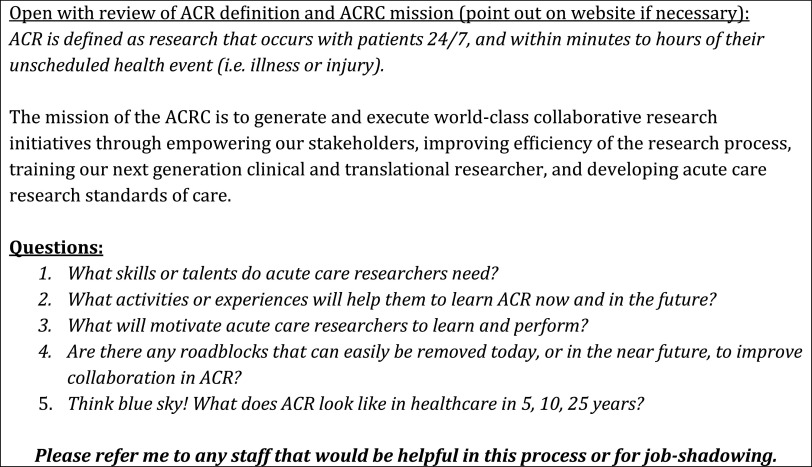
Semistructured interview guide.

Two data collection methods were utilized: semi-structured interviews and stakeholder observation. The principal researcher (SS) is a training specialist with experience in developing and facilitating professional development opportunities in higher education and industry. Interviews were conducted in person for approximately 30–60 min and followed the guide provided in Fig. 2. The interview questions were developed using Kirkpatrick’s problem-centered approach to adult experiential learning [9]. Interview data were documented during the interview by hand, and then post-interview data were entered in a de-identified spreadsheet organized by interview question. The research team maintained post-interview journals as well, to ensure that fresh insights or observations were captured immediately following an encounter with a participant [10]. These journals were not coded with the primary dataset but were instead used to inform the coding process when questions arose.

The second method of data collection was direct observation. The principal researcher (SS) observed participants in their clinical research setting, such as the ED or ICU, research office, or a training session. Care was taken to observe behaviors, skills, and knowledge that were called upon consistently during the participants’ work, without altering their environment [11]. Direct observation is a valuable method within qualitative research because it can provide unique knowledge of actual behavior in a specific setting in real time [12]. Extensive descriptive field notes were written down during observation in the observer’s notebook, followed up with post-observation reflective notes, both of which were entered in a de-identified spreadsheet.

### Data Analysis

A multiphase process was used to analyze the qualitative data, with the first step being data condensation and coding [8]. Data condensation was informed by both frequency of statement and emphasis by the participants. First-cycle codes were applied to high-level data chunks. As first-cycle codes crystallized about halfway through the data collection process, pattern codes naturally emerged, which are part of the second-cycle coding process [8]. Data saturation occurred after approximately three quarters of the interviews were completed [8]. Initial themes were shared with participants for feedback (member checking) and ACR characteristics, and themes were refined based on feedback.

In collaboration with the ACRC, the research team subsequently applied the principles of Bloom’s Taxonomy to generate a second draft, which more deliberately employed language consistent with a framework of educational goal setting [13]. Each competency was discussed to determine the level of mastery required for work proficiency, and the language was refined to reflect the agreed-upon level. Lastly, input received during member checking was again used to produce the final version of the ACR-CRP competencies.

## Results

In the first stage of recruitment, 22 interviews were conducted with members who were initially identified by the ACRC and related Advisory Councils. An additional 26 interviews were conducted from our snowball sample, in the second stage of recruitment, achieving a broadly representative sample. Additionally, 60 h of direct observation of ACR professionals in the clinical and research office setting were conducted. A total of 48 individuals participated in the study between February and May 2017, all 48 via interview and 11 via both interview and observation. The breakdown of the participants by identified strata is provided in Table 1 for both interview participants and observation participants. VAMC resources were not available for this study. Table 2 includes demographic details such as gender, education level, and years in current position, when available.

**Table 1.** Interview and observation participants by institutional affiliation and title

Academic role/job title	Interview participants by institutional affiliation		Observation hours by institutional affiliation	
	UCMC	CCHMC	UCMC	CCHMC
Principal Investigators				
Epidemiologist	2	1		
Pharmacist	1			
Medical Director (MD, PhD)	7	10		
Compliance Specialist	1			
Clinical Research Professionals				
Clinical Research Director	2	1		
Clinical Research Manager	6	4		
CRC I*	5	1	34	
CRC II	1	1	3	5
CRC III	2	1	5	2
CRC IV		1		2
Social Worker I		1		1
Research RN; Regulatory Specialist				4
Regulatory Specialist			2	
Trauma Bay (MDs/RNs/CRCs) *acute care trial simulation event*				2
Total	27	21	44	16

CCHMC, Cincinnati Children’s Hospital Medical Center; CRC, Clinical Research Coordinator; RN, registered nurse; UCMC, University of Cincinnati Medical Center.

* CRC levels reflect university appointment based on years of experience and expertise with higher levels representing more experience.

**Table 2.** Interview and observation participants demographics

Participant demographics	Principal investigators	Clinical research professionals	Total
Gender			
Male	13	5	18 (38%)
Female	7	23	30 (62%)
Highest educational level			
Less than a bachelor’s		2	2 (4%)
Bachelor’s or RN		14	14 (29%)
Master’s		11	11 (23%)
Doctorate	20	1	21 (44%)
Years of experience in clinical and translational research			
1 year or less		4	4 (8%)
2–4 years	2	5	7 (15%)
5–10 years	3	10	13 (27%)
10 years or more	15	9	24 (50%)

RN, registered nurse.

The interview dataset provided foundational material for a first draft of ACR-specific competencies. For example, communication and interpersonal skills specific to the ACR setting came up repeatedly. As one Clinical Research Director described, ACR-CRPs need to communicate concisely and articulately but also need to quickly build rapport with patients and care providers due to the fast-paced nature of ACR study procedures. Similarly, a Clinical Research Manager emphasized the need to be calm under pressure and to think quickly and creatively as patients’ state of health and care plan can change rapidly. Empathy was another characteristic that many participants stated to be important in ACR-CRPs since they are often interacting with patients and patients’ families in the midst of a traumatic health situation.

The multistep analysis process described above yielded an initial first draft of 28 ACR-specific competencies categorized under the existing eight JTF scientific domains. The next iteration of data analysis to this draft was then performed, leading to several types of changes within the results. Statements that were not actionable or measurable were transformed to align with Bloom’s spectrum of basic knowledge, more complete comprehension, application, analysis, synthesis of elements, and evaluation of materials or methods [13]. For example, the first draft of competency 23 under Study and Site Management originally read as an instruction: “Practice! Make sure you are competent on enrollment procedures and the operation of various equipment in order to deliver excellent task performance. Ask questions if you are unsure.” After applying Bloom’s Taxonomy and refining the specific capability that should be achieved within this area, this competency was rewritten in the final draft: “Exemplifies onsite preparedness at all times, in real time, ensuring proficiency on enrollment procedures and operation of various equipment in order to deliver excellent study task performance.”

**Fig. 3.**
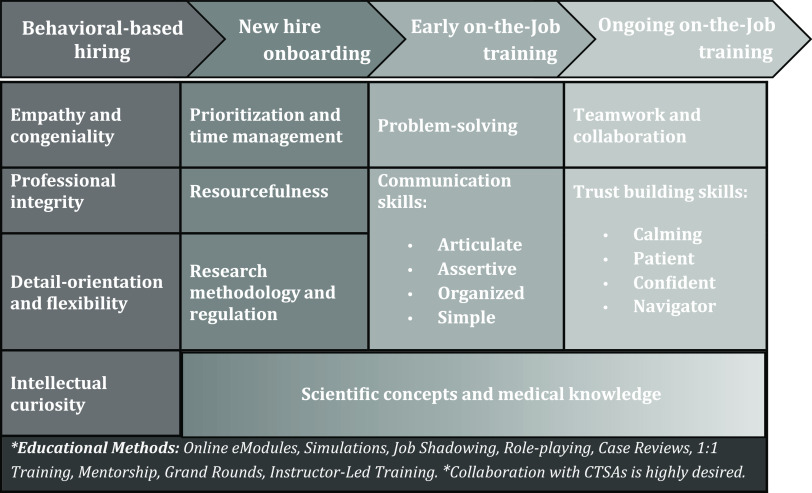
Acute care research (ACR)-CRP recruitment and professional development plan.

The JTF Harmonized Core Competency Framework – Version 2.0 lists eight competency domains with no priority order, emphasized by presenting them in a circle. For ACR-CRPs, however, member checking identified Communication and Teamwork, Clinical Study Operations (good clinical practices), and Data Management and Informatics as the highest priority skills. Table 3 presents the final competencies. These are in addition to skills already listed in the JTF’s core competencies, such as medical terminology and pathophysiology. Because many of the competencies described below are not just knowledge-based, they will function most effectively within a larger recruitment and professional development framework, described in the Discussion and presented in Fig. 3.

**Table 3.** Final ACR-CRP special interest competencies under re-prioritized JTF domains

Domain 1: Communication and teamwork
1. Understands each acute audience as its own vulnerable population and is considerate and empathetic of the diverse perspectives and feelings of participants.
2. Utilizes regular checkpoints in concise presentations to ensure common understanding and relatability and assesses appropriate audience comprehension and engagement, making no assumptions with participants nor ACR Team.
3. Anticipates needs from each enrollment presentation and adapts in response to the participants’ questions and feedback and proactively offers to gather disease-related questions to partner with MD/principal investigator.
4. Builds teamwork and trust with open and collaborative exchange of information among ACR Team and key stakeholders (e.g., IRB, RNs).
Domain 2: Clinical study operations (GCPs)
5. Models how to conduct oneself in an ethical manner, complying with acute care regulations, rules, and policies for the involved division(s), institution(s), and ICH-GCPs.
6. Utilizes open, patient, and constructive communication in emergency settings to breed a welcoming atmosphere of information updates to policies and procedures for the involved division(s) and institution(s).
7. Examines and adjusts, when appropriate, the strengths and weaknesses, costs and benefits, and short- and long-term consequences of multiple approaches and standards of care.
Domain 3: Data management and informatics
8. Designs data collection techniques in collaboration with the ACR Team that are user-friendly, succinct, and can be quickly executed correctly in the fast-paced acute setting and flexes ability to use basic math and problem-solving skills at any time.
9. Examines data in detail-oriented and accurate manner to ensure important gaps in existing information are eliminated, assures the integrity of the research data, and streamlines processes per ICH, FDA CFR Part 11.
10. Models efficiency, using tools to maximize amount of automated data entry, minimizing duplication and error as time is of the essence.
Domain 4: Ethical and participant safety considerations
11. Demonstrates empathy and a high level of understanding of this vulnerable patient population, in order to protect participants rights, as participation in acute care research is voluntary.
12. Determines participants’ capacity and ability to consent, recognizing when to best approach for ACR studies to maximize enrollment while minimizing stress and maintaining participants’ autonomy in research decisions.
13. Employs positive relationship building skills, using clarifying and confirming communication in presenting key information, halting in the face of uncertainty and being adaptable in the emergency setting.
14. Takes responsibility for one’s actions, admitting mistakes and treating them as learning experiences to ensure highest level of safety standards for acute (and all) participants.
Domain 5: Leadership and professionalism
15. Demonstrates ownership and confidence in the protocol, takes every opportunity to lead and mentor, and acts with integrity and patience in emergency situations.
16. Adapts well, flexible, and receptive to feedback and information.
17. Gathers alternatives to various difficult issues, troubleshooting, problem-solving, and determining judgment calls; sharing learnings will improve the creativity and collaboration within ACR Team.
18. Proactive about career development opportunities to advance education and anticipation of research partner needs (e.g., clinician test, blood draw, and regulatory document); taking lead to author research protocol(s) and paper(s).
Domain 6: Scientific concepts and research design
19. Exhibits intellectual curiosity for medical and research knowledge, even striving to be principal (or co/sub) investigator and/or co-author of research paper(s).
20. Fosters teamwork with acute care PIs and key ACR team partners to build an innovative ACR team science environment.
21. Creates professional development opportunities that broaden knowledge and skills to facilitate innovation in acute care research.
Domain 7: Study and site management
22. Envisions the work process from start to finish, meticulously ensuring eligibility criteria and study protocol adherence, including 24/7 timeframes and serious events reporting plan (e.g., blood draws required every 3 hours; pharmacy orders for drug infusions).
23. Exemplifies onsite preparedness at all times, in real time, ensuring proficiency on consenting, enrollment procedures and operation of various equipment in order to deliver excellent study task performance. (To achieve, must also have the support of tools and efficiencies in place.)
24. Cultivates relationships with key ACR stakeholders and decision makers who have the ability to provide needed hospital access, resources, information, and/or expertise (e.g., ACR-CRP access during/for overnight clinical events).
Domain 8: Medicines development and regulation
25. Demonstrates expertise and continuous pursuit of knowledge around new regulations impacting ACR, proactively sharing knowledge with ACR Team.
26. Prioritizes multiple studies appropriately, managing the IRB/FDA/Hospital/other regulatory bodies’ (e.g., HRPO) policies and procedures, in tandem and in a timely manner, for acute regulatory responsibilities.
27. Utilizes the *Exception from Informed Consent* for emergency research interventions under carefully controlled circumstances, ensuring required follow-up necessary once the intervention has begun.
28. Develops and monitors study protocol guidance for consenting vulnerable populations, specifically the requirements of a waiver of informed consent for a minimal risk study versus a study with greater than minimal risk, where a waiver cannot apply.

ACR, acute care research; CFR, Code of Federal Regulations; CRP, clinical research professional; FDA, Food & Drug Administration; GCP, good clinical practice; HRPO, Human Research Protection Office; ICH, International Council for Harmonisation; IRB, institutional review board; RN, registered nurse.

## Discussion

Through an iterative refinement process with participants and other key stakeholders involved in ACR, a novel set of special interest competencies for ACR-CRPs was developed. The framework was organized around the original eight domains found in the JTF Harmonized Core Competency Framework [1,4], although they were reordered to reflect the ACR priorities emphasized in the data captured, specifically Communication and Teamwork, Clinical Trials Operations, and Data Management and Informatics. The ACR-CRP competencies are not intended to replace the JTF Harmonized Core Competency Framework but instead build upon it and augment the development of a specialized ACR workforce through strategic hiring, focused onboarding programs, and early and ongoing on the job training, as discussed below and represented in Fig. 3. The advancement of CRP talent in ACR would aid in all aspects of the conduct of ACR trials and could strengthen the field of ACR by ensuring capable research staff are prepared to address critical ACR trials designed to improve the care and outcomes of critically ill and injured patients.

One novel discovery of this study was the need to place the ACR-CRP competency framework in a larger recruitment and professional development plan as illustrated in Fig. 3. This plan begins with behavioral-based hiring, which will help ensure that individuals who embody behaviors complementary to ACR-CRP job duties are hired from the start. Not only will the individuals hired have greater potential for job satisfaction in the ACR setting, but they are also likely to achieve job competency faster and more efficiently. Once hired, the professional development plan outlines standardized onboarding that provides ACR-CRPs with baseline knowledge of prioritization of projects, managing time around work duties, the resources available to support study management, and research methodology and regulation. Following completion of onboarding, ACR-CRPs will then undertake on-the-job training in two phases: early and ongoing. Early on-the-job training will introduce advanced problem-solving techniques and communication skills. Training on difficult skills such as building trust and working effectively in a team will be ongoing. Scientific concepts and medical knowledge will be incorporated throughout the professional development plan, and a variety of innovative training methods such as simulations, role-playing, and case reviews should be utilized to encourage active, applied learning.

A key interpretation of the iterative analysis process is that several unique behavioral attributes are expected of ACR-CRPs. While every effort was made to structure our ACR special interest competencies as skills that can be trained and mastered, iterative analysis of interview and observational data revealed that certain personality traits or characteristics are particularly vital in an ACR atmosphere that operates under critical time restrictions with a vulnerable patient population. Some examples of this include competencies that address proactive anticipation of needs (ACR competency 1.3), empathy and understanding (4.11), confidence and responsibility (4.14, 5.15), adaptability (5.16), intellectual curiosity (6.19, 8.25), and meticulous attention to detail (7.22). This led to the placement of the ACR competency framework within a larger workforce development plan that includes behavioral-based recruitment and professional development. Fig. 3 provides a general outline for how this plan could be structured, including the types of educational methods that could be utilized to achieve competency over time. Matching the behavioral traits to a career track became the central starting point of the plan, followed by supporting CRPs with ongoing training over multiple phases: onboarding, early on-the-job, and ongoing on-the-job training.

The ADDIE model prescribes next steps for the ACR competency framework: *Implement* and *Evaluate*. Implementation and evaluation will both require collaboration and organization across units and institutions to leverage strengths and expertise. ACR units will need to work together to organize existing training, identify training gaps, and develop new training materials to fill those gaps. Wherever possible, new hire onboarding and on-job training should be homogenized across hospitals, both to ensure standardization of procedures and to reduce institutional burden in the creation of materials and processes. Professional development should also be tied to the CRP career ladder in order to leverage meaningful participation with tangible benefits to the learners.

*Evaluation* is intended to focus on evaluating the competency framework itself. The evaluation should certainly include an evaluation of the competency of the learners operating within the framework, but that is only one piece. Several measures exist to assess CRP competency, both in general and in specialized roles such as data management and regulatory affairs [14,15]. These could be incorporated into an evaluation plan, as well as institutional metrics directly tied to clinical research activities (e.g., time from notice of grant award to study opening) [16] and CRP job satisfaction (including recruitment and retention).

This study has several strengths and limitations. Its major strengths lie in the study design and analysis. First, the study sample is comprehensive and includes support staff at all levels as well as principal investigators. Inclusion of CRPs across multiple strata of experience and affiliation was essential to developing competencies that are generalizable across a broad array of ACR staff from different institutions and different academic units. All participants had multiple opportunities to contribute to development of the competencies, and the final framework reflects this inclusive process. Second, the analysis process was iterative, entailing numerous phases of modification and refinement. Data interpretation took 6 months, and applying Bloom’s Taxonomy to desired characteristics, transformed behaviors and attitudes into measurable competencies that can support a professional development agenda. Perhaps most importantly, member checking occurred regularly during data analysis, increasing the credibility of our final results. Study limitations include a sample limited to the Cincinnati Academic Health Center, although this weakness is mitigated somewhat by inclusion of participants from multiple institutions and backgrounds. It is common practice in competency development to “level” or provide hierarchies of competence for each proficiency, such as the JTF accomplished, but this fell outside the aims of this study and could be a goal of future research.

By inviting ACR staff to contribute to this competency development project as both participants and member checks, our goal was to craft competencies that can guide the development of valuable, practical training, and professional development opportunities. The CRP segment of the workforce is an historically overlooked audience for training and professional development, but national efforts to support the staff that are so vital to the success of the clinical research enterprise are finally coalescing with some urgency. As research hospitals increasingly recognize that partnerships between EDs and ICUs are a critical foundation for ACR success, common competencies such as the ACR framework proposed here could greatly facilitate such collaborations. In a specialized field such as ACR, where patient vulnerability combined with care and time constraints are a daily reality, a well-trained workforce is particularly crucial. It is our hope that the ACR competencies outlined here and placed in a context of intentional recruitment and professional development will help to better prepare the ACR workforce of the future for the challenges – and rewards – of the field.

## Supplementary material

For supplementary material accompanying this paper visit https://doi.org/10.1017/cts.2020.38. click here to view supplementary material
