# Supplementary material for: Acute care research competencies for clinical research professionals
Source: J Clin Transl Sci. 2020 Apr 13;4(6):485–92. doi: 10.1017/cts.2020.38 (PMC8057453; doi:10.1017/cts.2020.38)
Supplement: Supplementary file 1 [file S2059866120000382sup001.docx]

# Appendix: 2017-2018 Advisory Groups

## Acute Care Research Council (ACRC)

***ACRC Co-Founders***

| Brett M. Kissela, MD, MS | UC Neurology/Stroke Team |
| --- | --- |
| Christopher J. Lindsell, PhD | UC Health Office of Clinical Research |

UC Emergency Medicine

***ACRC Co-Directors***

Opeolu Adeoye, MD, MS UC Emergency Medicine, Stroke Team

Lynn Babcock, MD, MS CCHMC Emergency Medicine
Timothy Pritts, MD, PhD UC Trauma/Surgery, Critical Care

***ACRC Partners***

| Richard Becker, MD, MS | UC Cardiovascular Health & Disease |
| --- | --- |
| Richard Falcone, MD, MPH | CCHMC Trauma Services |
| Brandon Foreman, MD | UC Neurocritical Care |
| Robert Frenck, MD | CCHMC IRB |
| Stuart L. Goldstein, MD | CCHMC Center for Acute Care Nephrology |
| Dina Gomaa, BS, RRT, CCRC | UC Trauma, Critical Care |
| Michael Linke, PhD | UC IRB |
| Eric Mueller, PharmD | UC Health Investigative Pharmacy |
| Laura Ngwenya, MD, PhD | UC Neurotrauma Center |
| Katrina Peariso, MD, PhD | CCHMC Neurology |
| Brenda Poindexter, MD, MS | CCHMC Perinatal Institute |
| Richard Ruddy, MD | CCHMC Emergency Medicine |
| David Russell, JD | CCHMC Perinatal Institute |
| Misty Troutt, BS, MS, MBA | CCHMC Trauma/Surgery |
| Hector Wong, MD | CCHMC Critical Care |

## Acute Care Research – Clinical Research Professionals (ACR-CRP) *also members of Acute Care Research Regulatory Professionals (ACR-RP)

***ACR-CRP Partners***

| Dina Gomaa, BS, RRT, CCRC | | Clinical Research Director, UC Trauma, Critical Care | |
| --- | --- | --- | --- |
| Rebecca Ingledue, CCRP | | CRC IV, UC Heart, Lung & Vascular Institute (UC HLVI) | |
| Sara Keegan, MEd | | Clinical Research Manager, UC Emergency Medicine | |
| Cassie Kirby, BA, CCRP* | | CRP IV, CCHMC Center for Acute Care Nephrology | |
| Carol Knochelmann, RN | | Research Nurse, UC HLVI | |
| Kelli Krallman RN, MS, CCRC, ACRP-PM* | | Research Nurse, CCHMC Center for Acute Care Nephrology | |
| Samantha Kramer, BS* | | CRC IV, UC Nephrology | |
| Stacey Liddy-Hicks, MS | | Clinical Research Director, CCHMC Emergency Medicine | |
| Omar Lopez, BS* | | CRC II, UC Neurosurgery/Neurocritical Care | |
| Rachael Mardis, RN | | Research Nurse, UC HLVI | |
| Angela Molloy, RN, BSN, CCRP* | | Clinical Research Project Manager, UC Neurology | |
| Tammy Roads, CCRP | | Clinical Research Director, UC HLVI | |
| Carolina Rodriguez, BA, CCRP* | | Regulatory Manager, UC Trauma/Surgery | |
| Susan Roll, RN, BSN, CCRP* | | Central IRB Liaison, UC StrokeNet | |
| David Russell, JD, CIP, CHRC* | | Regulatory, CCHMC Neonatology/Perinatal Institute | |
| Dorice Smith, BA, CPhT | | Pharm Tech IV, UC Health Investigative Drug Services | |
| Cristina Spinner, BS, CCRP* | | Regulatory Specialist, UC Emergency Medicine | |
| Lara Stone, MA* | | CRP IV/Regulatory, CCHMC Emergency Medicine | |
| Autumn Studer | | Clinical Research Associate, UC HLVI | |
| Misty Troutt, BS, MS, MBA | | Clinical Research Director, CCHMC Trauma/Surgery | |
